# Exogenous Tryptophan Promotes Cutaneous Wound Healing of Chronically Stressed Mice through Inhibition of TNF-α and IDO Activation

**DOI:** 10.1371/journal.pone.0128439

**Published:** 2015-06-09

**Authors:** Luana Graziella Bandeira, Beatriz Salari Bortolot, Matheus Jorand Cecatto, Andréa Monte-Alto-Costa, Bruna Romana-Souza

**Affiliations:** Laboratory of Tissue Repair, Department of Histology and Embryology, State University of Rio de Janeiro, Rio de Janeiro, Brazil; University Hospital of Heidelberg, GERMANY

## Abstract

Stress prolongs the inflammatory response compromising the dermal reconstruction and wound closure. Acute stress-induced inflammation increases indoleamine 2, 3-dioxygenase-stimulated tryptophan catabolism. To investigate the role of indoleamine 2, 3-dioxygenase expression and tryptophan administration in adverse effects of stress on cutaneous wound healing, mice were submitted to chronic restraint stress and treated with tryptophan daily until euthanasia. Excisional lesions were created on each mouse and 5 or 7 days later, the lesions were analyzed. In addition, murine skin fibroblasts were exposed to elevated epinephrine levels plus tryptophan, and fibroblast activity was evaluated. Tryptophan administration reversed the reduction of the plasma tryptophan levels and the increase in the plasma normetanephrine levels induced by stress 5 and 7 days after wounding. Five days after wounding, stress-induced increase in the protein levels of tumor necrosis factor-α and indoleamine 2, 3-dioxygenase, and this was inhibited by tryptophan. Stress-induced increase in the lipid peroxidation and the amount of the neutrophils, macrophages and T cells number was reversed by tryptophan 5 days after wounding. Tryptophan administration inhibited the reduction of myofibroblast density, collagen deposition, re-epithelialization and wound contraction induced by stress 5 days after wounding. In dermal fibroblast culture, the tryptophan administration increased the cell migration and AKT phosphorylation in cells treated with high epinephrine levels. In conclusion, tryptophan-induced reduction of inflammatory response and indoleamine 2, 3-dioxygenase expression may have accelerated cutaneous wound healing of chronically stressed mice.

## Introduction

Stress is the phenomenon in which a potentially harmful stimulus results in physiological or psychological disturbances to homeostasis [1]. Chronic stress may compromise cutaneous wound healing through prolonged inflammatory response induced by high levels of catecholamines and glucocorticoids [2–6]. An increased inflammatory response blunt the immune response and delays the development of subsequent phases of wound healing impairing wound closure leading to increase in the susceptibility to infections and patients’ mobility [7–10]. Several studies have investigated the mechanisms by which stress-induced inflammation compromises the cutaneous wound healing. Recently, it was proposed that suppressive and pro-inflammatory effects of stress may be related to indoleamine 2, 3-dioxygenase (IDO)-mediated tryptophan catabolism [11].

Tryptophan, an essential amino acid, is the precursor of neurotransmitter serotonin [5-hydroxytryptamine (5-HT)] which regulates gastrointestinal functions, mood and appetite [12, 13]. Tryptophan catabolism is mediated by tryptophan dioxygenase activation mainly in the liver or by IDO inducible by bacterial products (as lipopolysaccharide) and pro-inflammatory mediators [as tumor necrosis factor-α (TNF-α) and interferon-γ (IFN-γ)] in several tissues [12, 13]. Both enzymes are able to catalyze the conversion of tryptophan into kynurenine, quinolinic acid and kynurenic acid through a complex metabolic pathway [13]. Increased depletion of tryptophan via tryptophan dioxygenase pathway increases the production of kynurenines which inhibit T cell responses and cause the development of dendritic cells with tolerogenic properties [14]. A diet deficient in tryptophan increases plasma corticosterone levels and reduces plasma 5-HT levels and 5-HT positive cells in the dorsal and median raphe of chronically stressed rats [15]. Patients submitted to peri-operative stress presents an increase in the kynurenine/tryptophan ration which might contribute to the increased risk of infection and postoperative immunosuppressive states [11]. In addition, the increase in the tryptophan catabolism and serotonin degradation is related to schizophrenia and major depression in human [16]. Thus, some studies have proposed that the L-tryptophan administration may be an alternative method to reverse the effect of stress on immune system and behavior.

Firstly it was proposed that the dietary supplementation with tryptophan associated to anti-depressives could attenuate the depressive disturbs in stressed patients [17, 18]. Carbohydrate-rich diets may raise the plasma tryptophan levels and decrease depressive mood and plasma cortisol levels during acute laboratory stress in humans [19, 20]. Diets with high levels of tryptophan diminish plasma concentrations of cortisol and noradrenaline and enhance the recovery after social stress in pigs [21]. In mice, the treatment with L-tryptophan may have an anti-inflammatory action reducing pancreatitis, stomach ulceration and contact hypersensitivity through increased production of interleukin-10 and decreased synthesis of nuclear factor kappa-light-chain-enhancer [22–24]. Thus, anti-inflammatory action of tryptophan administration may be helpful to reduce the immunosuppressive effects of stress improving cutaneous wound healing.

Therefore, the aim of this study was to investigate the effect of L-tryptophan administration on cutaneous wound healing in chronically stressed mice. In addition, this study evaluated the effects of high epinephrine concentrations and L-tryptophan on murine skin fibroblast cultures. We show that tryptophan administration inhibits stress-induced IDO activation and skin inflammation accelerating the cutaneous wound healing of mice. We also demonstrate that tryptophan directly reverses the impairment in the migration of murine dermal fibroblasts through AKT phosphorylation.

## Material and Methods

### Animals

Male Swiss mice with 3 months of age were kept in groups (5 animals per cage) under controlled conditions with 12 hour light/dark cycle. All procedures were carried out in strict accordance with the Brazilian Legislation regarding Animal Experimentation (no 11.794, from October 8, 2008). All experiments in this study were approved by the Ethical Committee for Animal Use of the State University of Rio de Janeiro (Permit Number: 027/2014). All surgery was performed under ketamine and xylazine anesthesia, and all efforts were made to minimize suffering.

### Restraint stress

Animals (n = 40) were daily restrained in well-ventilated, plastic and cylindrical tubes [10.5 cm (height) x 4.5 cm (internal diameter)] for 1 hour per day, until euthanasia. Mice did not have access to food or water while in the tubes. Nonstressed groups (n = 40) were deprived food and water during the same time period, although they were free to roam in their cages [25]. Restraint stress was performed between 9:00 a.m. and 10:00 a.m.

### Administration of L-tryptophan

From 1 day after the beginning of the stress protocol, a group of stressed and nonstressed animals was intraperitoneally treated with 50 mg/kg of L-tryptophan (Sigma-Aldrich, St. Louis, MO) dissolved in 0.9% saline and 0.1N hydrochloric acid (9:1), daily until euthanasia. Another group of stressed and nonstressed animals was treated only with vehicle. The administration of L-tryptophan or vehicle was performed after restraint stress between 9 a.m. and 10 a.m. Study dose was adapted from previous study where rats with experimental ulcers received 100 mg/kg/day of L-tryptophan [23, 26]. Thus, mice were divided into four groups as follows (20 animals per group): *nonstressed group*: animals not stressed that received only vehicle; *nonstressed+tryptophan group*: animals not stressed that received tryptophan; *stressed group*: animals stressed that received only vehicle; *stressed+tryptophan group*: animals stressed that received tryptophan. During the study, all mice were fed with standard chow containing 157 mg of tryptophan per 100 g of chow (PragSoluções Bioscience, São Paulo, Brazil) ad libitum and had free access to water.

### Wound model

Two days after the beginning of the stress protocol, all animals were intraperitoneally anesthetized with ketamine (150 mg/kg) and xylazine (15 mg/kg). After shaving the dorsum, two circular full-thickness excisional wounds were created using a biopsy punch with 8 mm diameter. The lesions were separated by a bridge of normal skin and located 2 cm from the occipital bone of the cranium. The wounds were not sutured or covered and healed by secondary intention.

### Macroscopic analysis

To evaluate wound contraction, lesions were measured soon after wounding and after 3, 5 and 7 days later without scab removal as described [27]. The results were expressed as percentage of the original wound area. To evaluate re-epithelialization, the margins of the total wound area and non-re-epithelialized wound area were measured 7 days after wounding as described [27]. The results were expressed as percentage of the re-epithelialized wound area.

### Tissue harvesting and microscopic analysis

One hour after the tryptophan or vehicle injection, mice (10 animals per day) were intraperitoneally anesthetized with ketamine (150 mg/kg) and xylazine (15 mg/kg) 5 or 7 days after wounding. One hour after anesthesia, mice were decapitated and the peripheral blood was collected and plasma was frozen at -70°C. Thereafter, lesions and adjacent normal skin samples of five animals per group were collected, and these tissue samples were formalin-fixed (pH 7.2) and paraffin-embedded. For each group, lesions of five animals per group were collected and frozen at -70°C. Frozen lesions were macerated in lysis buffer and total protein concentration was determined using the bicinchoninic acid protein assay (Thermo Fisher Scientific, Rockwood, TN). This lysate was used to perform lipid peroxide levels, ELISA and immunoblotting.

Sections (5 μm thick) were stained with hematoxylin-eosin to measure migratory tongue length. For this, slides were digitalized using Pannoramic Digital Slide Scanner (3DHistech Ltd., Budapest, Hungary). The migratory tongue length was measured using Pannoramic Viewer software (3DHistech Ltd., Budapest, Hungary). Length was defined as the distance (in micrometers) from the wound edge to the tip of the tongue as described [28]. The results were presented in μm.

### Immunohistochemistry and quantification

Immunohistochemistry was used to investigate the amount of neutrophils (myeloperoxidase), macrophages (F4/80) and T cells (CD-3). The following antibodies were used: rat monoclonal to myeloperoxidase (#71674; Santa Cruz Biotechnology, Santa Cruz, CA; 1:500), rat monoclonal to F4/80 (#497; Serotec Inc., Raleigh, NC; 1:500) and rabbit polyclonal to CD-3 (#5690; Abcam, Boston, MA; 1:100) as previously described [28, 29]. To quantify the number of immunostained cells, five random fields per animal (14,689 μm^2^) were analyzed as previously described [28]. The results were presented as cells per mm^2^. To observe the distribution of serotonin receptor-7 (SR-7)-positive cells in skin and wound area of nonstressed mice, sections were also incubated with rabbit polyclonal antibody to SR-7 (#28963; Santa Cruz Biotechnology; 1:500).

Quantification of myofibroblasts was performed using sections immunolabelled with mouse monoclonal antibody to α-smooth muscle actin (#0851; DAKO, Carpinteria, CA; 1:100) plus anti-mouse EnVision System (#4001; DAKO; 1:20) as previously described [30]. Volume density of myofibroblasts was evaluated using point counting, x40 objective lens and videomicroscopic system as previously described [28, 31, 32]. The results were presented as volume density of myofibroblasts (Vv_[myofibroblasts]_%).

### Immunoblotting

Proteins were separated by sodium dodecylsulfate-polyacrylamide, transferred to polyvinylidene fluoride membrane and probed with antibodies: rabbit polyclonal to IDO (45 KDa) (#106134; Abcam; 1:50), rabbit polyclonal to SR-7 (43–45 KDa) (#28963; Santa Cruz Biotechnology; 1:200), goat polyclonal to monocyte chemotactic protein-1 (MCP-1) (12 KDa) (#1784; Santa Cruz Biotechnology; 1:200), rabbit polyclonal to active transforming growth factor (TGF-β)-1/2/3 (17 KDa) (#7892; Santa Cruz Biotechnology; 1:200), mouse monoclonal to collagen type III (138 KDa) (#3392; Chemicon International, Inc., Temecula, CA; 1:600), mouse polyclonal to collagen type I (140–210 KDa) (#765; Chemicon International, Inc.; 1:500) or mouse monoclonal to β-actin (42 KDa) (#5441; Sigma-Aldrich; 1:1000). Following incubation with the appropriate horseradish peroxidase-conjugated secondary antibodies, immune complexes were detected using enhanced chemiluminescence (Santa Cruz Biotechnology). The β-actin was used as a loading control protein and the results were expressed as arbitrary units.

### Enzyme-linked immunosorbent assays (ELISA)

The protein levels of TNF-α were measured using an ELISA assay (#555268; BD Biosciences Pharmingen, San Diego, CA). The assay was performed according to the manufacturers’ instructions. The results were presented as pg TNF-α per mg total protein.

### Biochemical analyses

To confirm the stress-induced physiological alterations, norepinephrine synthesis was indirectly estimated by assaying the plasma levels of normetanephrine, which is a major and stable metabolic end-product of norepinephrine, as previously described [2, 33]. The results were expressed as ng/μl of normetanephrine.

To confirm the efficiency of L-tryptophan administration, the plasma levels of L-tryptophan were determined in wound lysate using a colorimetric assay as described [34]. The results were expressed as mg/ml of L-tryptophan.

To evaluate the lipid peroxidation, the levels of lipid peroxides were measured in wound lysate using the ferrous oxidation-xylenol orange method as previously described [35]. The results were expressed as nmol lipid peroxides per μg total protein.

### Behavior evaluation

Another experiment was performed using the same stress protocol to examine locomotor activity and anxiety-like behavior. For this, a group of animals (n = 5) was evaluated before and after 8 days of restraint stress (corresponding to 5 days after wounding). These animals were not treated with L-tryptophan or vehicle and did not receive cutaneous lesions.

In the open-field test, the animals were placed in the center of a white plastic cage (40 x 33 x 17 cm) containing 16 rectangles (9.5 x 8.5 cm). The number of the covered rectangles for each animal was counted for 5 minutes [2]. For analysis, a Sony video-camera and monitor (Sony, United Kingdom) were used. These results were expressed number of rectangles per minute.

In the elevated plus-maze, the animals were placed on the central platform of the plus-maze consisted of two open and two closed arms (10 cm wide x 50 cm long, 50 cm walls for closed, 1 cm walls for open) elevated 40 cm off the floor (Insight, São Paulo, Brazil). Each animal was tested for 5 minutes on the maze. The percentage of time spent in open arms provided as the measures of anxiety was calculated for each animal [36].

### Cell culture

Primary fibroblasts were isolated from the skin of nonstressed Swiss mice (1–2 months) by the standard explant technique as previously described [6, 37]. The cells were routinely maintained in Dulbecco’s modified Eagle’s medium (DMEM) (Sigma-Aldrich) supplemented with 10% fetal bovine serum (FBS) (Cultilab Ltda, Campinas, Brazil) and antibiotics (100 UI/ml penicillin, 50 μg/ml kanamycin, 100 μg/ml streptomycin, and 6 μg/ml amphotericin B) (Sigma-Aldrich) at 37°C and 5% CO_2_. Cells were seeded for experiments when they reached about 95% confluence. Experiments were performed at passages 3 to 10, at least three times, in triplicate.

In all experiments, cells were treated with epinephrine (100 μM) (Hipolabor, Minas Gerais, Brazil), L-tryptophan (10 μM) (Sigma-Aldrich), or epinephrine plus L-tryptophan [38]. The dose of 10 μM of L-tryptophan was based on a dose-dependent and time-dependent study (data not shown) and previous study [39]. Drugs were prepared in DMEM with 2% FBS and antibiotics.

### Cell viability assay

Cell viability was determined using the 3-(4,5-dimethylthiazol-2-yl)-2,5-diphenyltetrazolium assay [6]. For this, fibroblasts (3 x 10^4^ cells/well) were treated and after 1, 2 or 3 days of treatment, 3-(4,5-dimethylthiazol-2-yl)-2,5-diphenyltetrazolium solution (Sigma-Aldrich) was added [6]. The results were expressed as the percentage of the control (medium-only) group.

### Cell migration assay

Cell migration was assessed by the ability of cells to move into an acellular area in a two-dimensional scratch assay [6]. Fibroblasts (2.5 x 10^5^ cells/well) were treated and a scratch was made in the center of the well with a 100 μl pipette tip. The denuded area was measured soon after scratching and at 1, 2 and 3 days later. The results were expressed as the percentage of the initial denuded area.

### Immunoblot for activation of AKT, IDO and SR-7

To evaluate the AKT phosphorylation, fibroblasts (5 x 10^5^ cells/well) were incubated in serum-free DMEM alone (control) or serum-free DMEM containing epinephrine or epinephrine plus L-tryptophan for 15 minutes. After treatment, cells were washed with ice-cold PBS and scraped in lysis buffer (20 mM Tris-HCl pH 7.5, 138 mM NaCl, 10% glycerol, 1% Triton X-100, protease inhibitor cocktail, 50 mM sodium fluorite, 1 mM sodium orthovanadate) (Sigma-Aldrich). Proteins were resolved by sodium dodecylsulfate-polyacrylamide, transferred to polyvinylidene fluoride membrane and immunoblotted with rabbit polyclonal antibodies for Akt1/2/3 (#8312) or phospho-AKT 1/2/3 Ser 473 (#33437; Santa Cruz Biotechnology; 1:200) as described [38]. To confirm the protein expression of IDO and SR-7 in dermal fibroblast culture, cells (5 x 10^5^ cells/well) in DMEM with 2% FBS and were scraped in lysis buffer. The protein levels of IDO and SR-7 were estimated in protein extract using western immunoblot protocol as described above. The results were expressed as arbitrary units.

### Statistical analysis

All data is presented as mean ± standard error of the mean (SEM). Statistical analysis was performed by using one-way ANOVA followed by Bonferroni`s post-test or unpaired Student’s t-test. The values of p<0.05 were considered statistically significant for all tests. GraphPad Prism software was used to perform the statistical analyses (GraphPad Prism version 5.0, San Diego, CA).

## Results

### L-tryptophan administration on restrained stress-induced physiological alterations

**Fig 1:**
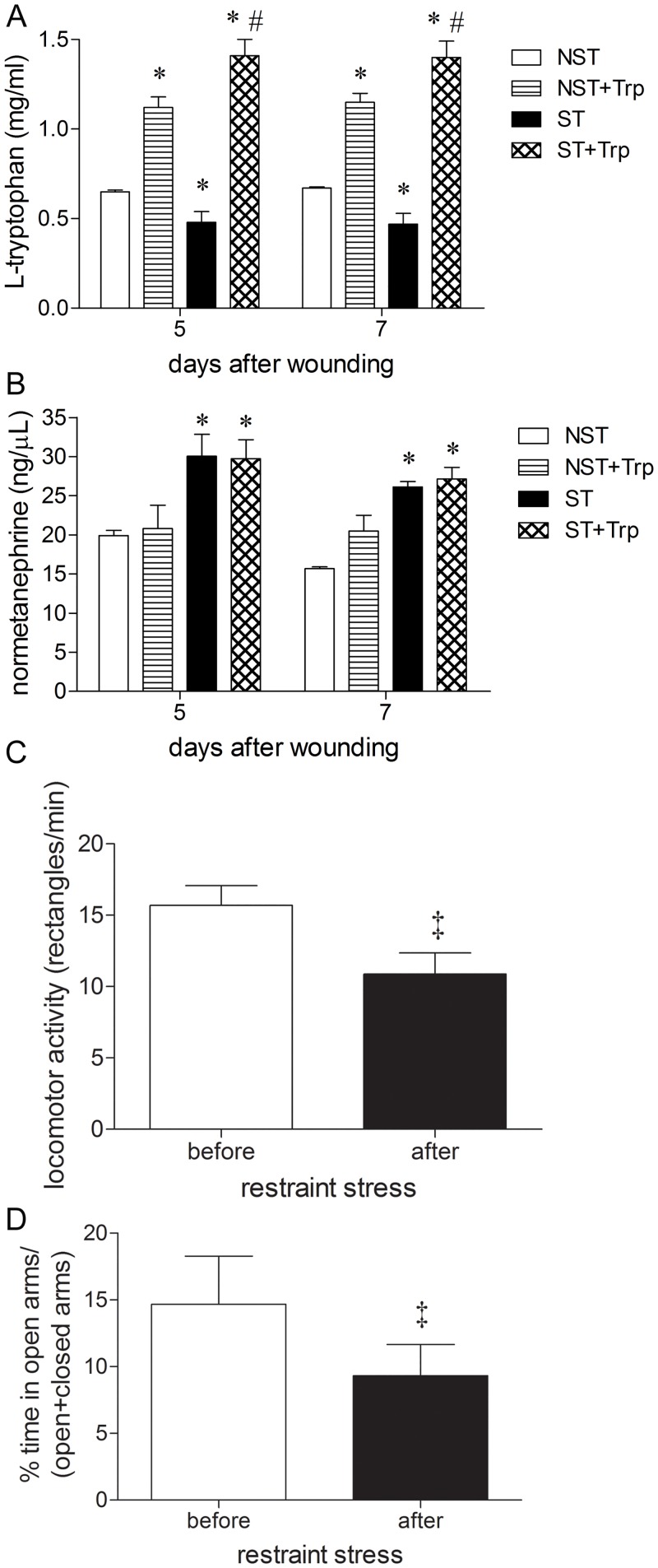
Evaluation of L-tryptophan administration and stress model. Mice were daily submitted to restraint stress and treated with L-tryptophan (Trp) or vehicle until euthanasia. Nonstressed mice were not subjected to the stress protocol or treated with L-tryptophan. Two days after the beginning of the stress protocol, two full-thickness excisional lesions (8-mm diameter) were made on the dorsal skin. (A) Plasma levels of L-tryptophan were measured in nonstressed and stressed groups 5 and 7 days after wounding. (B) Physiological alterations induced by restrained stress were evaluated through plasma levels of normetanephrine 5 and 7 days after wounding. (C) Locomotor activity was evaluated before and after induction of restraint stress using open field test. (D) Anxiety-like behavior was measured before and after induction of restraint stress using elevated plus-maze. These results were expressed as percentage time spent in open arms per time spent in open and closed arms. The animals submitted to behavior evaluations (C, D) were not treated with L-tryptophan or received cutaneous lesions. Data (n = 5) are presented as mean ± SEM. *p<0.05 vs. nonstressed (NST) group. ^#^p<0.05 vs. stressed (ST) group. ^‡^p<0.05 vs. animals before the beginning of the restrained stress.

Stressed group presented a slight reduction in the plasma levels of L-tryptophan when compared with nonstressed group 5 and 7 days after wounding (Fig 1A). Two hours after intraperitoneal injection of L-tryptophan (50 mg/kg) there was a 1.5–3.0 fold increase in plasma L-tryptophan concentration in the nonstressed and stressed groups when compared to vehicle-injected groups 5 and 7 days of wounding (Fig 1A). Nonetheless, L-tryptophan administration did not alter stress-induced increase in the plasma levels of normetanephrine 5 and 7 days after wounding when compared with nonstressed group (Fig 1B). There was no difference in plasma normetanephrine levels between nonstressed and nonstressed+tryptophan groups 5 and 7 days of wounding (Fig 1B). To confirm behavioral alterations induced by stress, the locomotor activity and anxiety-like behavior were evaluated. After 8 days (corresponding to 5 days after wounding), restrained stress reduced the locomotor activity and induced anxiety-like behavior in mice (Fig 1C and 1D).

### L-tryptophan administration reduces stress-induced tryptophan catabolism through IDO and TNF-α

**Fig 2:**
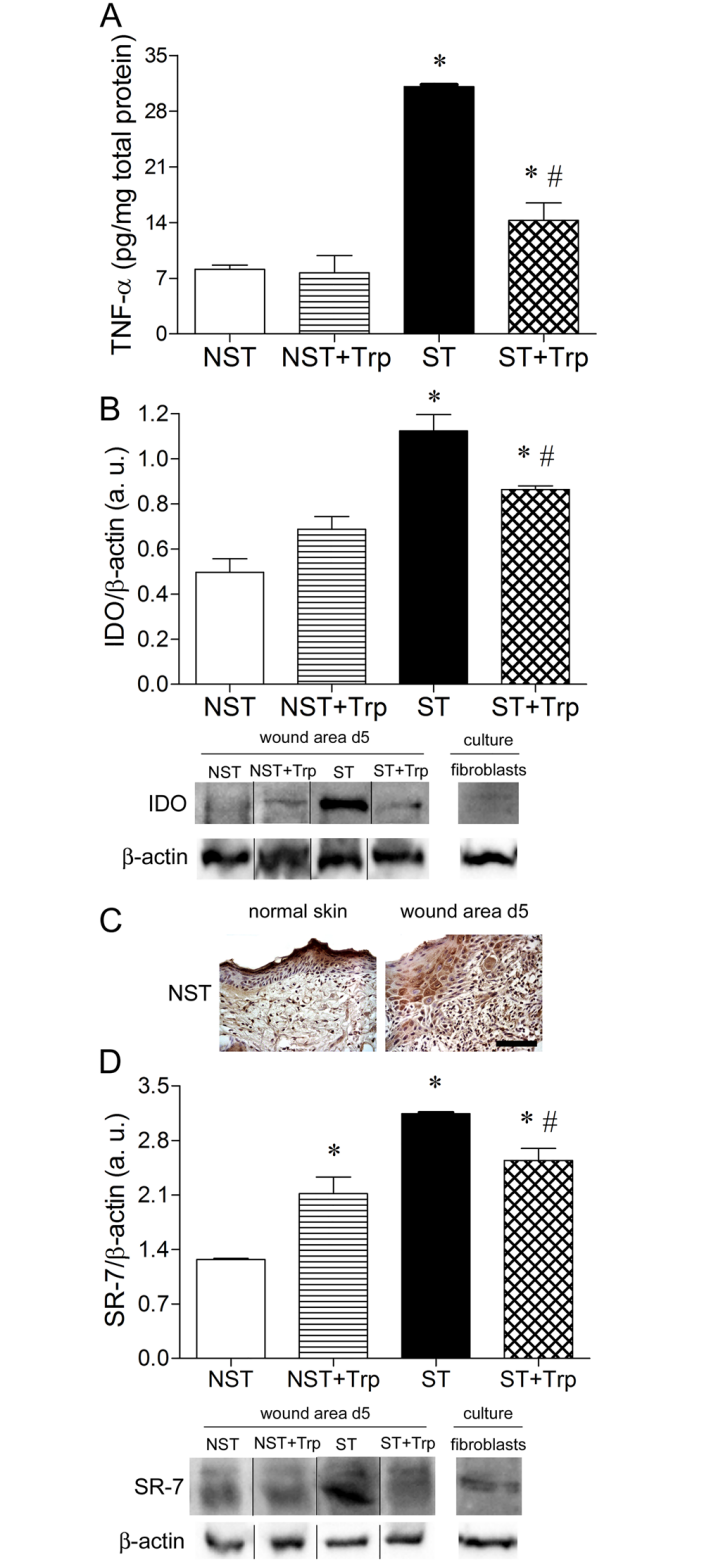
L-tryptophan administration reverses the stress-induced alterations on tryptophan catabolism. Mice were daily submitted to restraint stress and treated with L-tryptophan (Trp) or vehicle until euthanasia. Two days after the beginning of the stress protocol, two full-thickness excisional lesions (8-mm diameter) were made on the dorsal skin. Five days after wounding, lesions were lysated and the protein levels of tumor necrosis factor-α (TNF-α) (A) were measured by ELISA assay. The protein levels of indoleamine 2,3-dioxygenase (IDO) (45KDa) (B) were measured by immunoblotting. The expression of the IDO (B) was also detected in murine skin fibroblast culture. To observe the SR-7 expression on normal skin and wound area, immunohistochemistry for SR-7 (C) was performed in paraffin-embedded section of normal skin and wound area at 5 days (d5) of the mice which were not stressed or treated with L-tryptophan. Bar = 50 μm. In addition, the protein levels of serotonin receptor-7 (SR-7) (43–45 KDa) (D) were measured by immunoblotting. The expression of the SR-7 (C) was also detected in murine skin fibroblast culture. The β-actin (42 KDa) was used as a loading control protein. Vertical black lines show non-adjacent bands from the same blot. Data (n = 5) are presented as mean ± SEM. ^#^p<0.05 vs. stressed (ST) group.

Psychological stress increases the protein expression of TNF-α in wounded area of mice when compared to nonstressed mice [2, 3, 5]. To investigate the effects of psychological stress and L-tryptophan administration on TNF-α synthesis, the protein levels of TNF-α were measured on wound area of studied groups through ELISA assay. L-tryptophan administration attenuated the stress-induced increase in the protein levels of TNF-α in wound area when compared to nonstressed group 5 days after wounding (Fig 2A). There was no difference in TNF-α protein levels between nonstressed and nonstressed+tryptophan groups 5 days of wounding (Fig 2A). Recently it was demonstrated that pro-inflammatory response to stress is involved to tryptophan catabolism through interferon-γ, TNF-α and IDO induction in mice brain [11]. We verified if L-tryptophan administration could reduce IDO protein levels in wound area of stressed mice. Five days after wounding, restrained stress increased protein levels of IDO in wound area when compared to nonstressed group (Fig 2B). The L-tryptophan administration attenuated the effect of stress on IDO protein expression 5 days after wounding (Fig 2B). There was no difference in IDO protein expression between nonstressed and nonstressed+tryptophan groups 5 days of wounding (Fig 2B). We also observed that skin fibroblasts incubated in medium alone expressed IDO proteins (Fig 2B). Acute stress-stimulated induction of IDO in mice may decrease the plasma levels of tryptophan and serotonin [11]. To investigate the effect of psychological stress and L-tryptophan administration on skin regulation by serotonin, SR-7 protein expression was detected. Initially, we demonstrated that SR-7 was expressed in normal skin and wound area of mice which were not submitted to stress or received tryptophan. For this, the immunostaining for SR-7 were performed in normal skin and wound area of mice. The SR-7 staining was observed in keratinocytes (granular layer) and fibroblastic-like cells of wound area and normal skin of mice which were not submitted to stress model or received tryptophan (Fig 2C). In addition, skin fibroblasts incubated in medium alone also expressed SR-7 protein (Fig 2D). The protein levels of SR-7 were greater in the stressed group than in the nonstressed group 5 days after wounding (Fig 2D). However, L-tryptophan administration reduced the effect of stress on SR-7 protein expression (Fig 2D). There was no difference in SR-7 protein expression between nonstressed and nonstressed+tryptophan groups (Fig 2D).

### Stress-stimulated wound inflammation and lipid peroxidation are reduced by L-tryptophan administration

**Fig 3:**
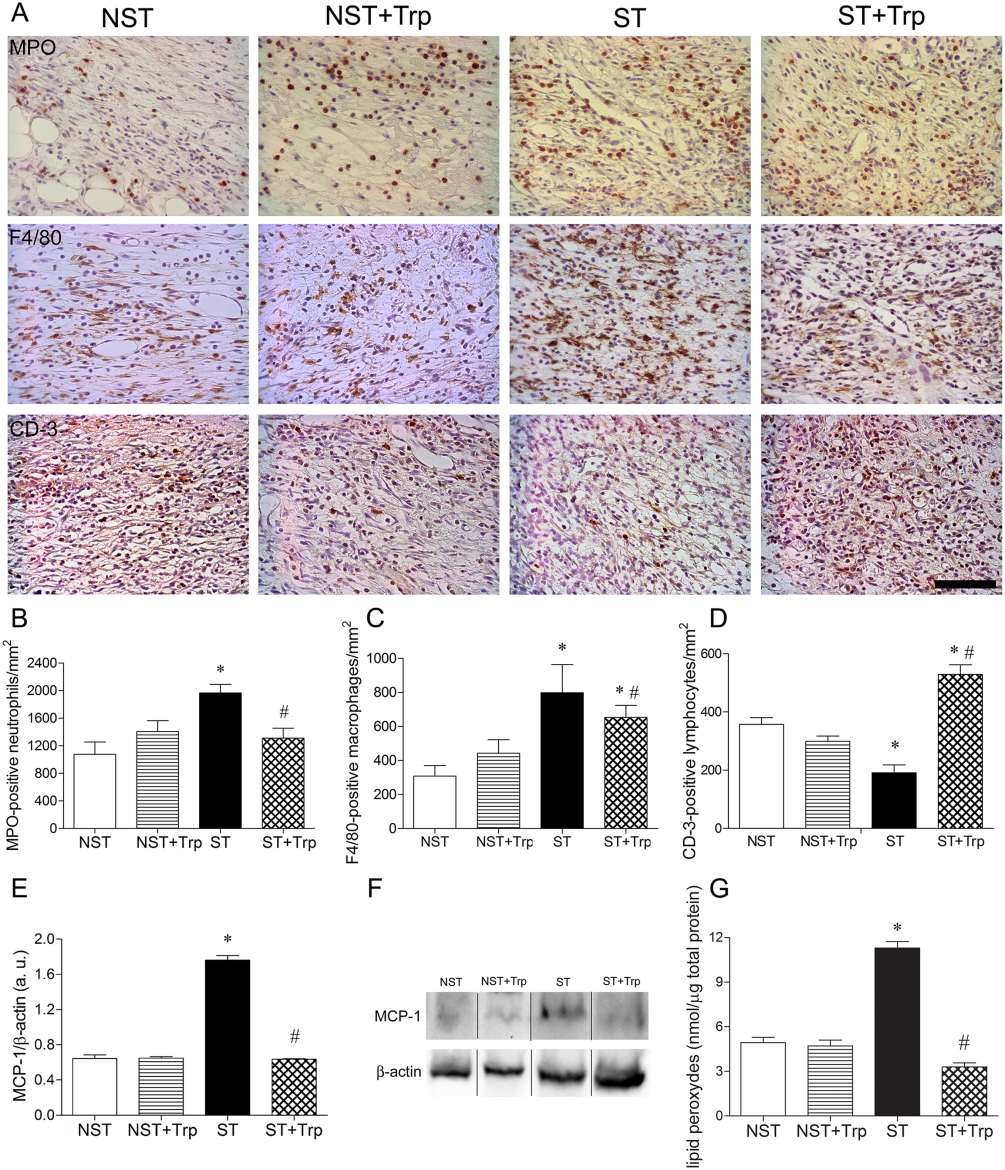
L-tryptophan administration reverses stress-induced alterations on inflammatory response and lipid peroxidation. Mice were daily submitted to restraint stress and treated with L-tryptophan (Trp) or vehicle until euthanasia. Two days after the beginning of the stress protocol, two full-thickness excisional lesions (8-mm diameter) were made on the dorsal skin. Five days after wounding, lesions were collected and the number of myeloperoxidase (MPO)-positive neutrophils (B), F4/80-positive macrophages (C) and CD-3 positive T lymphocytes (D) were counted. Representative images (A) of staining for neutrophils, macrophages and T cells on wound area were presented. In addition, the protein levels of monocyte chemotactic protein-1 (MCP-1) (12 KDa) (E) were estimated in wound lysate by western-blotting. Representative images for immunoblotting for MCP-1 (F) were presented. The β-actin (42 KDa) was used as a loading control protein. Vertical black lines show non-adjacent bands from the same blot. To evaluate the oxidative damage in lipids, the levels of lipid peroxides (G) were measured in wound lysate using colorimetric assay. Data (n = 5) are presented as mean ± SEM. ^#^p<0.05 vs. stressed (ST) group. Bar = 50 μm.

Psychological stress increases the neutrophil and macrophage migration and lipid peroxidation in the wound area of mice when compared to nonstressed mice [2, 3, 6]. To determine if L-tryptophan administration could reverse the stress-induced increase in wound inflammation and oxidative damage, the inflammatory response and lipid peroxidation were evaluated. Five days after wounding, the number of neutrophils and macrophages was greater in the stressed group than in the nonstressed group (Fig 3A–3C). In addition, the number of neutrophils and macrophages was reduced in stressed animals after L-tryptophan administration (Fig 3A–3C). The number of CD-3 positive T cells was smaller in the stressed group than in the nonstressed group 5 days after wounding (Fig 3D). L-tryptophan administration increased the CD-3-positive T cells number on wound area of nonstressed and stressed mice 5 days after wounding (Fig 3D). There was no difference in neutrophil, macrophage and T cells number between nonstressed and nonstressed+tryptophan groups (Fig 3A–3D). The MCP-1 is a potent factor of the inflammatory response in cutaneous lesions which recruits macrophages and induces the subsequent production of TNF-α [40]. Stress increased the MCP-1 protein levels and lipid peroxide levels when compared to nonstressed group 5 days after wounding (Fig 3E–3G). The administration of L-tryptophan decreased the protein levels of MCP-1 on wound area of stressed mice 5 days after wounding (Fig 3E and 3F). Stress-induced oxidative damage in lipids (lipid peroxide levels) was reversed by L-tryptophan administration 5 days after wounding (Fig 3G). There was no difference in MCP-1 protein expression and lipid peroxides levels between nonstressed and nonstressed+tryptophan groups (Fig 3E–3G).

### Stress-induced impairment in the dermal reconstruction and wound closure is reversed by L-tryptophan administration

**Fig 4:**
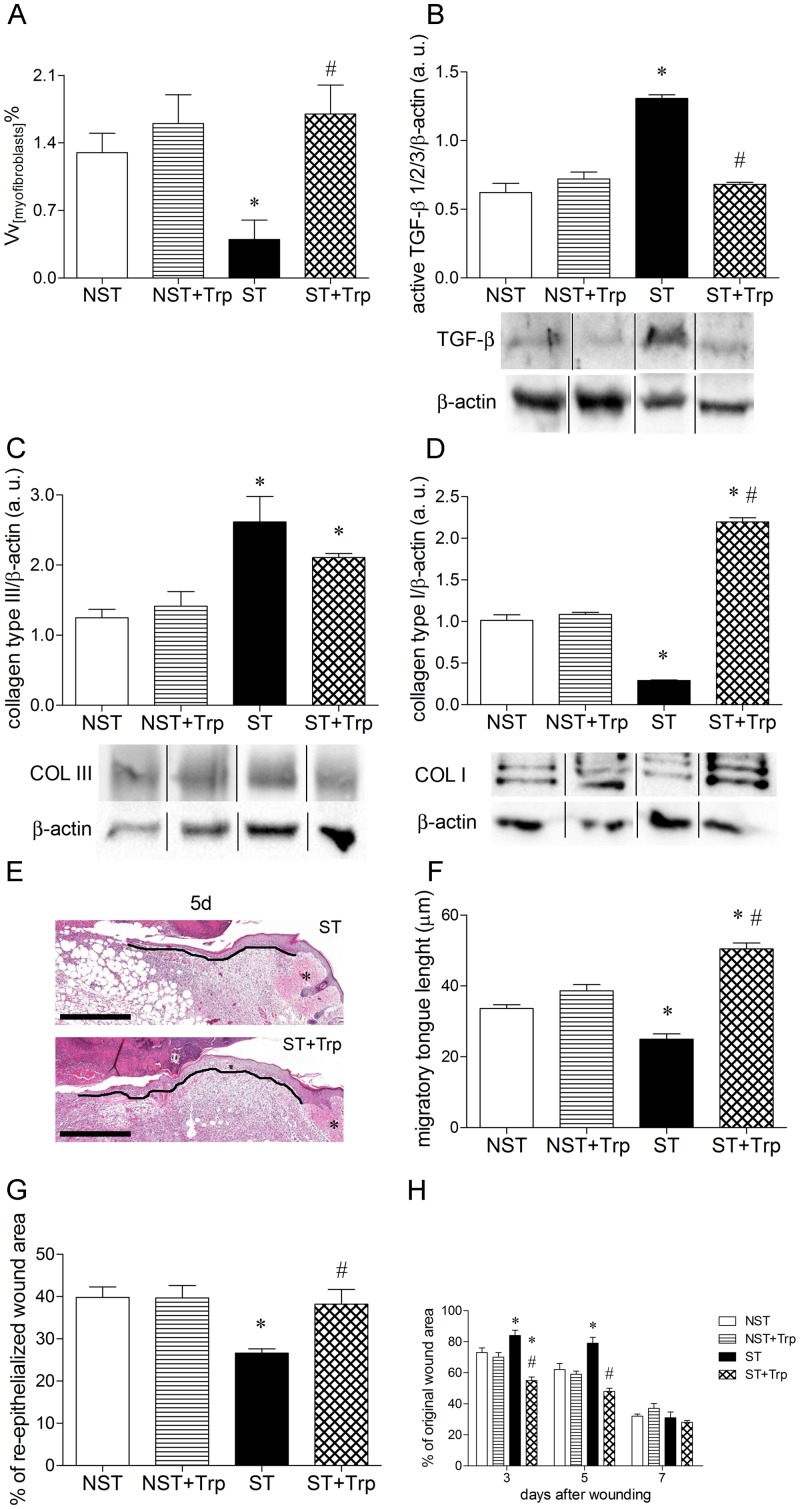
L-tryptophan administration improves the dermal reconstruction in wound area of stressed mice. Mice were daily submitted to restraint stress and treated with L-tryptophan (Trp) or vehicle until euthanasia. Two days after the beginning of the stress protocol, two full-thickness excisional lesions (8-mm diameter) were made on the dorsal skin and collected five or seven days after wounding. To evaluate the myofibroblastic differentiation, the volume density of myofibroblast (Vv_[myofibroblast]_%) (A) and the protein levels of active transforming growth factor-β (TGF-β) 1/2/3 (17 KDa) (B) were measured in wound lysate 5 days after wounding. The β-actin (42 KDa) was used as a loading control protein. Vertical black lines show non-adjacent bands from the same blot. To measure the collagen deposition, the protein levels of collagen type III (COL III) (138 KDa) (C) at 5 days and collagen type I (COL I) (140–210 KDa) (D) at 7 days were measured in wound lysate. The β-actin (42 KDa) was used as a loading control protein. Representative images of migratory tongue (E) in stressed (ST) and stressed+tryptophan (ST+Trp) groups after five (5d) days of wounding. Black dotted line shows migratory tongue length (E), asterisk show normal skin (E) and sections are stained with hematoxylin-eosin. Bar = 500 μm. To evaluate the neo-epidermis formation, the length of migratory tongue (F) at 5 days and the percentage of re-epithelialized wound area (G) at 7 days were measured. To evaluate the wound contraction, the percentage of original wound area (H) was measured 3, 5 and 7 days after wounding. Data (n = 5 or n = 10) are presented as mean ± SEM. ^#^p<0.05 vs. stressed (ST) group.

Psychological stress increases myofibroblast density and protein TGF-β expression, but reduces collagen deposition, re-epithelialization and wound contraction when compared to nonstressed mice [2, 3, 5, 6]. To investigate the effect of L-tryptophan administration on dermal reconstruction and wound closure of the stressed mice, myofibroblastic differentiation, collagen deposition, re-epithelialization and wound contraction were evaluated. Stress reduced the myofibroblast density, but increased the active TGF-β protein levels when compared to nonstressed group 5 days after wounding (Fig 4A and 4B). The L-tryptophan administration increased myofibroblast density, but reduced the protein levels of active TGF-β 1/2/3, in wound area of stressed mice 5 days after wounding (Fig 4A and 4B). Stress increased the collagen type III protein levels, but reduced the collagen type I protein levels, when compared to nonstressed group at 5 days (Fig 4C). The L-tryptophan administration did not alter the effect of chronic stress in the protein expression of collagen type III at 5 days (Fig 4C). However, the protein expression of collagen type I was increased in wound area of stressed mice by L-tryptophan administration 7 days after wounding (Fig 4D). Stress decreased the migratory tongue length, the re-epithelialization and wound contraction when compared to nonstressed group (Fig 4E–4H). The L-tryptophan administration reversed stress-induced impairment in the migratory tongue length at 5 days and re-epithelialization at 7 days (Fig 4F and 4G). In addition, L-tryptophan administration inhibited the stress-induced delay in the wound contraction 3 and 5 days after wounding (Fig 4H). There was no difference in myofibroblast density, TGF-β protein levels, collagen type I and III, migratory tongue length, re-epithelialization and wound contraction between nonstressed and nonstressed+tryptophan groups (Fig 3A–3G).

### L-tryptophan inhibits high epinephrine levels-induced impairment in the fibroblast migration

**Fig 5:**
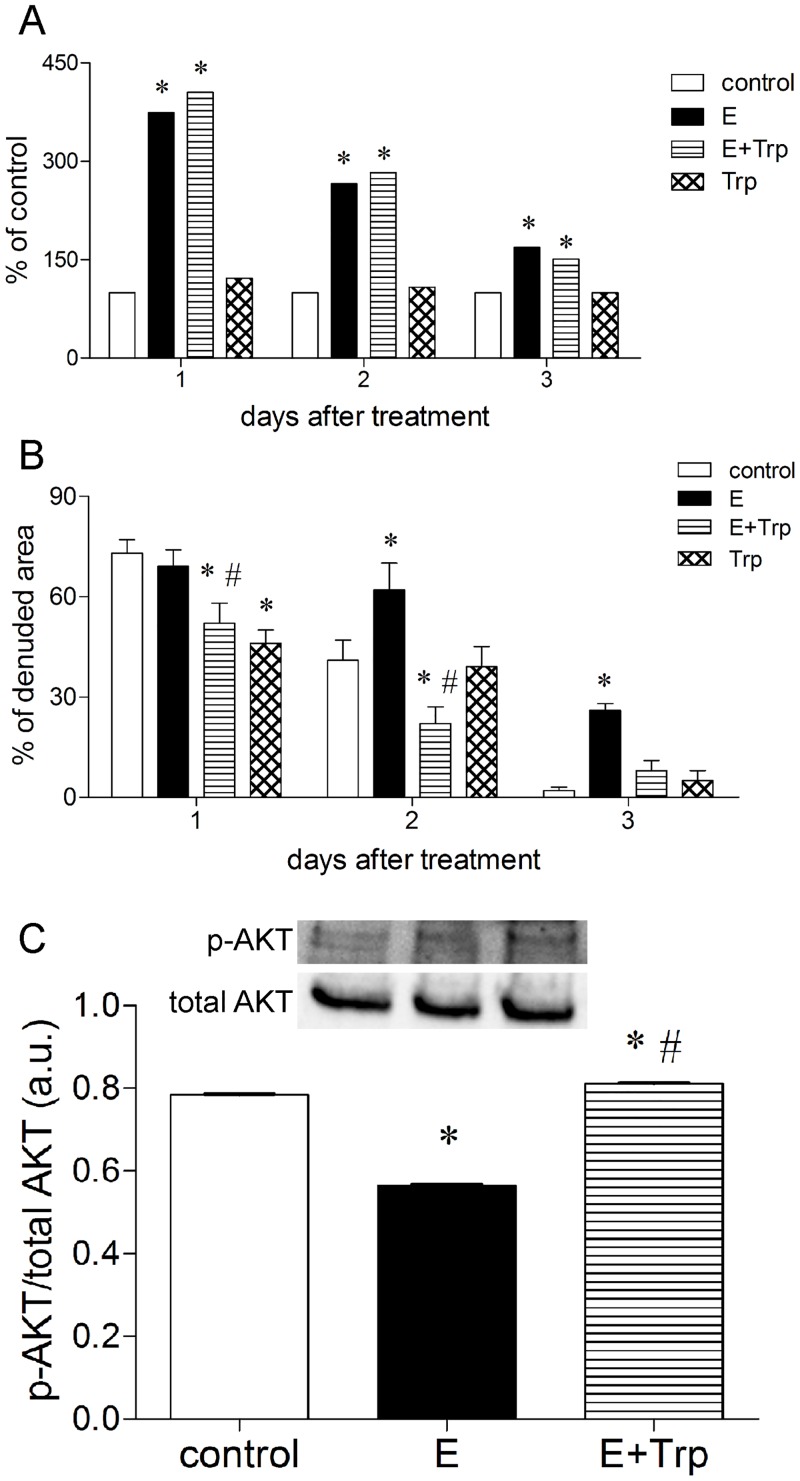
Tryptophan administration inhibits epinephrine-induced impairment in the dermal fibroblast migration through AKT phosphorylation . Murine skin fibroblasts were plated in 24- or 96-well plates in triplicate and allowed to attach for 24 hours. Therefore, cells were treated with epinephrine (E) (100 μM), L-tryptophan (Trp), epinephrine plus L-tryptophan (E+Trp) (10 μM) or medium alone (control). To evaluate cell viability (A), cells were incubated in 3-(4,5-dimethylthiazol-2-yl)-2,5-diphenyltetrazolium solution and the formazan product was detected. To evaluate cell migration (B), cells were removed from an area by scratching and the denuded area was measured soon after scratching and 1, 2 and 3 days after later. Cell migration was expressed as a percentage of the initial denuded area (% of denuded area). To measure the protein expression of p-AKT^Ser 473^/total AKT (C), cell lysate was collected after 15 minutes of treatment; p-AKT/total-AKT ratio was measured by western-blotting and expressed in arbitrary units (a.u.). The data are representative of three independent experiments. Data (n = 9) are expressed as mean ± SEM. *p<0.05 vs. control cells (medium alone). ^#^p<0.05 compared to epinephrine (E).

High levels of epinephrine increases the proliferation of the murine skin fibroblast through ERK phosphorylation [2, 6, 38]. The L-tryptophan administration did not alter cell viability of fibroblasts treated with medium alone and high levels of epinephrine at 1, 2 and 3 days (Fig 5A). High levels of epinephrine reduced the migration of the murine dermal fibroblasts through AKT phosphorylation [38]. L-tryptophan administration inhibited the high epinephrine levels-induced delay in the dermal fibroblast migration after 1, 2 and 3 days of treatment (Fig 5B). To investigate if L-tryptophan administration could improve the fibroblast migration through AKT activation, the protein levels of p-AKT⁄total AKT was estimated. The L-tryptophan administration reversed epinephrine-induced reduction of the AKT phosphorylation at 15 minutes (Fig 5C).

## Discussion

Several studies have demonstrated that psychological stress impairs the cutaneous wound healing in humans, and rodent models [2–6, 9, 41, 42]. The primary mechanism by which stress compromises cutaneous wound healing is through prolonged inflammatory response induced by high levels of catecholamines and glucocorticoids. During the early phases of wound healing, psychological stress may suppress the infiltration of inflammatory cells and the synthesis of TNF-α through the immunosuppressive effects of norepinephrine and glucocorticoids [2]. Long-term exposure to norepinephrine and glucocorticoids leads to resistance in inflammatory cells (such as neutrophils and macrophages), thus inducing an increased inflammatory response and delaying the development of subsequent phases of wound healing [2]. Recently, it was demonstrated that acute stress induces a new inflammatory pathway which suppresses immune functions [11]. The acute stress-induced increase of TNF-α and IFN-γ synthesis activates the expression of mRNA IDO in the brain, lung and spleen of the mice increasing plasma levels of kynurenine and reducing the plasma levels of tryptophan and serotonin [11]. The derivates of tryptophan catabolism by IDO (as kynurenine and kynurenic acid) reduce T cell proliferation and the thickness of the thymic cortex of repeatedly stressed mice contributing to immunosuppressive effects and mood alterations [11, 43–45]. In depressed patients, the increase in the plasma TNF-α and IFN-γ levels may be associated to the reduction of the plasma tryptophan levels [46]. These observations prompted us to investigate whether the IDO-mediated tryptophan catabolism participates in adverse effects of stress on cutaneous wound healing. In addition to determine whether the inhibition of this pathway by tryptophan administration could promote the wound healing of stressed mice. To induce chronic psychological stress, animals were submitted to restrained stress model which is a well-defined psychological stress model with laboratory mice that compromises the animals’ housing leading to anxiety-like behavior, minor locomotor activity and excessive catecholamine synthesis [25, 36, 47, 48]. Previous study has demonstrated that human dermal fibroblasts express IDO mRNA and the activation of IDO by IFN-γ stimulates the tryptophan catabolism and kynurenine synthesis in *in vitro* studies [49]. In our study, the cutaneous lesions and skin fibroblasts of nonstressed mice expressed protein IDO and its expression is increased by chronic stress. In addition, restraint stress also decreased plasma tryptophan levels, but reduced the protein TNF-α levels in the wound area of mice. When stressed mice were treated with tryptophan not only there was a noticeable reduction of protein IDO and TNF-α levels, but also a strong increase in the plasma tryptophan levels. These results suggest that tryptophan administration may have inhibited the activation of IDO-mediated tryptophan catabolism on wound area contributing to reduction of the immunosuppressive and pro-inflammatory effects of chronic stress on cutaneous wound healing. This idea is reinforced by the increase in SR-7 protein levels on wound area of stressed mice. Mice skin expresses seven subtypes of SR (as SR-1A/1B and SR-7) in melanocytes and fibroblasts and is capable to produce serotonin [50, 51]. Repeatedly restraint stress reduces the serum and skin levels of 5-HT and the levels of SR-1A and SR-1B mRNA in mice skin, but increases SR-7 mRNA levels in mice submitted to chronic unpredictable stress [11, 51]. We believed that SR-7 protein expression in wound area of stressed mice may have been sensitive to the stress-stimulated reduction of serotonin levels. Therefore, SR-7 expression on wound area may be an additional evidence for the inhibitory effect of tryptophan on IDO-induced tryptophan catabolism pathway stimulated by stress.

Chronically stressed mice presented an exacerbate inflammatory response (as increased number of neutrophils, macrophages and T cells, and TNF-α protein levels) in wound area which was inhibited by tryptophan administration. These results are similar to previous studies where elevated levels of stress hormones prolong inflammatory responses compromising the development of its subsequent phases [2, 3, 5, 6]. Some studies demonstrate that stress-induced activation of IDO may reduce T cell proliferation and the thickness of thymic cortex in mice [11, 43]. In addition, GCN2 kinase phosphorylation via IDO induction may promote the proliferative arrest and anergy induction in mice T cells [44]. The supplementation with tryptophan may reverse IDO-mediated suppression of CD8^+^ dendritic cells isolated from murine tumor-draining lymph nodes [45]. Thus, we suggest that tryptophan-induced reduction of the TNF-α and IDO expression on wound area may have improved the local inflammatory response and promoted the dermal reconstruction and wound closure. Studies with gastric ulceration in rats demonstrate that tryptophan administration has anti-inflammatory action due to inhibition of nuclear factor kappa-light-chain-enhancer in activated B cells [23]. In our study, we also propose that anti-inflammatory effects of tryptophan may be related to MCP-1, a key molecule for chemotaxis and activation of macrophages [52], since tryptophan administration reversed the increase in MCP-1 protein levels in wound area of stressed mice. A surprising result was the tryptophan-induced reduction of the lipid peroxidation in wound area of stressed mice. Chronic stress-stimulated high levels of catecholamines increase the oxidative damage in lipids in mice cutaneous lesions and murine dermal fibroblast culture [6]. This observation may indicate that tryptophan administration may have an antioxidant effect on cutaneous wound repair of stressed mice for a mechanism is not still understood. Nonetheless, the antioxidant effects of tryptophan are reinforced by a previous study where a diet rich in tryptophan reduces the expression of mRNA for superoxide dismutase in the ulcerated mucosa of rats [23].

The adverse effects of chronic stress on dermal reconstruction and wound contraction are associated to two events: stress-induced prolonged skin inflammation and epinephrine-induced impairment of the skin fibroblast and keratinocyte activity [2–4, 6, 38, 42]. In addition, stress-induced catecholamines delay myofibroblastic differentiation, but reduce collagen deposition leading to the imbalance between myofibroblast-extracellular matrix and the impairment of the wound contraction [2, 6, 38]. Human keratinocyte migration is delayed by high levels of epinephrine through the reduction of the AKT phosphorylation [42]. In murine dermal fibroblasts, stress-induced epinephrine enhances myofibroblastic differentiation through increased expression of latent TGF-β 1/2/3, but decreases cell migration and collagen deposition [2, 6, 38]. In this study, we observed that tryptophan was capable to reverse the increase in the myofibroblastic differentiation and protein TGF-β levels, and the reduction of collagen type I deposition in wound area of stressed mice. In addition, tryptophan promoted keratinocyte migration (migratory tongue length) leading to increased re-epithelialization. We suggest that tryptophan-induced reduction of the local inflammatory response may be one of factors which leaded to acceleration of the dermal reconstruction, re-epithelialization and wound contraction. However, the positive effects of tryptophan on dermal reconstruction may be involved to dermal fibroblast activity. It has been demonstrated that high levels of epinephrine decreases the migration of murine dermal fibroblasts through the inhibition of the phosphatidylinositol 3-kinase (PI3K) ⁄AKT pathway. Tryptophan administration did not alter the viability of epinephrine-treated fibroblasts, but strongly inhibited the effects of epinephrine on fibroblast migration and AKT phosphorylation. These findings may be associated to the activation of the PI3K/AKT/mammalian target of rapamycin (mTOR) pathway. The activation of mTOR via PI3K/AKT pathway promotes the initiation of ribosomal translation which is essential to growth factor signaling [14, 53]. In T cells, the IDO induction decreases the free tryptophan which blocks the activation of mTOR contributing to immunosuppression [14]. Chronic stress decreases mTOR phosphorylation in the amygdala of rats through the reduction of AKT and extracellular signal-related kinase 1/2 activation [53]. Recently, it was demonstrated that PI3K/AKT activation promotes the proliferation and migration of keratinocytes and accelerates cutaneous wound healing of mice via mTOR [54]. Thus, the mechanism by which tryptophan inhibits the adverse effects of stress on dermal reconstruction and wound contraction is not only through the normalization of skin inflammation, but also through this direct effect on migration of dermal fibroblasts probably by the activation of PI3K/AKT/mTOR pathway.

## Conclusion

L-tryptophan administration improves the cutaneous wound healing of the chronically stressed mice probably by the inhibition of the TNF-α-induced activation of IDO. In mice, the reduction of the immunosuppressive and pro-inflammatory effects of chronic stress by tryptophan normalizes the inflammatory responses, decreases the lipid peroxidation and improves the myofibroblastic differentiation, collagen deposition, re-epithelialization and wound contraction. In addition, L-tryptophan administration promotes the migration of murine dermal fibroblasts exposed to high epinephrine level through the increase in the AKT phosphorylation.
